# Spontaneous splenic rupture mimicking pneumonia: a case report

**DOI:** 10.1186/1757-1626-1-35

**Published:** 2008-07-15

**Authors:** Karen A MacKenzie, Roy L Soiza

**Affiliations:** 1 Department of Medicine for the Elderly, Woodend Hospital, Aberdeen, AB15 6XS, UK; 2 Division of Applied Medicine, University of Aberdeen, Polwarth Building, Foresterhill, Aberdeen, AB25 2ZD, UK

## Abstract

A 74-year-old gentleman presented with a history of left-sided pleuritic chest and upper abdominal pain. Examination and chest x-ray findings were suggestive of pneumonia. An abdominal ultrasound was suggestive of spontaneous splenic rupture. An abdominal computed tomography scan showed a splenic laceration and large peri-splenic haematoma. The advice from the on-call surgical team was to treat conservatively but the patient's condition deteriorated suddenly and he died. Spontaneous splenic rupture is uncommon but probably under-diagnosed and should be considered in all patients presenting with non-specific abdominal pain. The optimal management strategy for the older patient with spontaneous ruptured spleen is unknown.

## Case Report

A 74-year-old Caucasian man presented with a one-week history of gradual onset, sharp epigastric and left-sided lower chest pain. The pain radiated to his left shoulder and was exacerbated by movement and coughing. The patient denied any history of trauma, foreign travel and was not on anticoagulants. His past medical history included mild ischaemic heart disease and depression. He was an active man, still in employment, who did not smoke or drink alcohol.

On examination he was tachycardic at 110 beats per minute, normotensive and had a temperature of 37.8°C. He was tachypnoeic with oxygen saturation of 91% on air. There was dullness to percussion and decreased air entry at the left base. Examination of the abdomen revealed mild generalised tenderness to palpation, worst in the epigastrium. There was no guarding, rebound tenderness or evidence of bruising.

Routine admission blood tests revealed a haemoglobin of 16.3 g/dl, a normal white cell count and C-reactive protein. His urea and creatinine were 19.1 mmol/l and 117 μmol/l respectively. His liver function tests were deranged with a bilirubin of 61 μmol/l, alanine aminotransferase 97 U/l, alkaline phosphatase 436 U/l and gamma glutamic transpeptidase 398 U/l. A chronic liver disease screen was normal, and there was no serological evidence of viral infection (including hepatitis and Epstein Barr virus).

Chest x-ray showed a left pleural effusion and consolidation. He was initially treated for pneumonia.

Chest and abdominal ultrasound scans showed a large amount of free fluid surrounding the spleen suggesting splenic rupture and a moderate left sided pleural effusion. The spleen was otherwise normal in size and appearance, and no other intra-abdominal abnormality was found.

He was referred to the surgeons who felt that the most appropriate line of management was conservative and suggested a follow-up ultrasound scan in four months time to monitor progress. Draining the pleural effusion was thought to be too risky due to its proximity to the haematoma.

**Figure 1:**
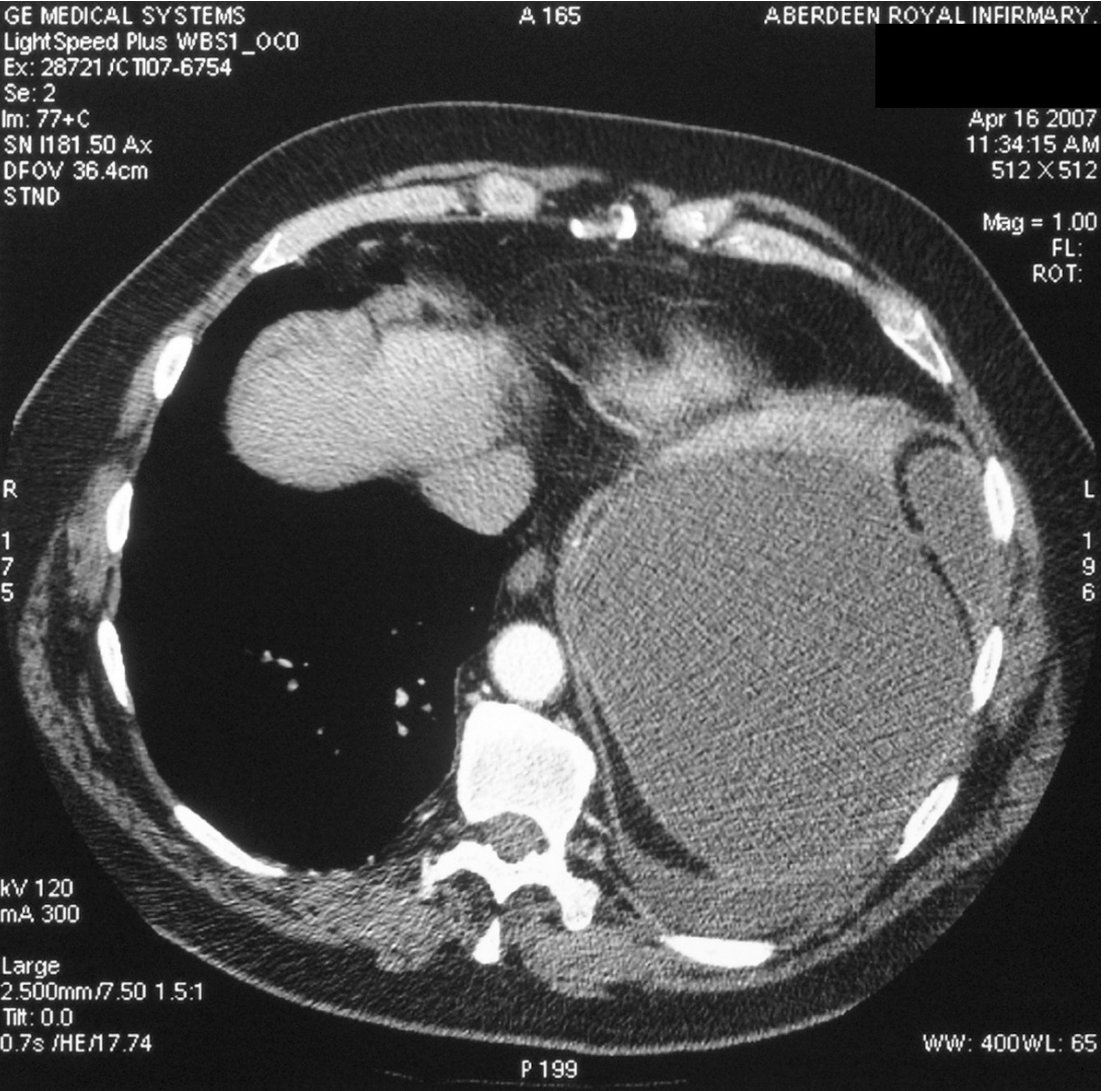
**Computed tomography scan**. Axial view of patient's CT scan showing a large peri-splenic haematoma.

During the subsequent week the patient developed pleuritic chest pain and a CT pulmonary angiogram and upper abdominal CT scan were performed. These showed no evidence of pulmonary embolism but revealed a splenic laceration with a peri-splenic subcapsular haematoma, measuring 16 × 14 cm in axial dimension (Figure 1), with a reactive pleural effusion. Once again, there was no other abnormality seen in the liver or spleen.

Unfortunately, before the patient could be re-reviewed regarding surgical intervention, he had an unwitnessed cardiac arrest and died. The family declined a post-mortem examination.

## Discussion

Rupture of the spleen is most commonly seen soon after blunt trauma to the abdomen or lower ribs. Iatrogenic splenic injuries are thought to be responsible for up to 40% of all splenectomies performed [1].

Spontaneous splenic rupture is uncommon but probably under-diagnosed in the absence of trauma [2]. Such splenic rupture may be classified as either "pathological" in the presence of underlying disease or "spontaneous" if normal [2,3]. There is debate as to whether this entity exists or whether rupture always occurs in the presence of undiagnosed disease.

Classically patients may present with symptoms of pain, tenderness and guarding of the left upper quadrant and hypovolaemic shock [4]. Left-shoulder tip pain, particularly when lying flat (Kehr's sign), is caused by blood irritating the left hemi-diaphragm and is said to be present in 50% of cases of splenic rupture [5]. However, symptoms can be subtle and the condition may be mistaken for angina pectoris, myocardial infarction, pulmonary embolism, peptic ulceration or, as in this case, pneumonia.

The management of spontaneous and traumatic splenic rupture is similar. In stable patients there is a relatively recent trend towards non-operative management often due to concerns regarding the risk of post-splenectomy infection [6,7]. A search of Medline, Embase and all Evidence Based Medicine Reviews combining several search terms for older people and spleen or splenic rupture failed to come up with any relevant articles to guide management. Similarly, a search for published guidelines on NHS Scotland e-library search facility did not reveal any specific guidelines for the management of spontaneous splenic rupture. The Society for Surgery of the Alimentary Tract recommend non-operative support with in-hospital observation for up to 5 days is adults with splenic injury and haemodynamic stability. Accepted indications for splenectomy in adults include haemodynamic instability, bleeding greater than 1000 ml, transfusion of more than 2 units of blood, or other evidence of ongoing blood loss [8]. However, these guidelines are chiefly based on traumatic splenic rupture in younger people. Since older people are known to have altered homeostatic mechanisms that make them more prone to haemodynamic collapse [9], it is unclear if more aggressive intervention may be warranted. Furthermore, concerns about rendering patients asplenic for decades are important only in younger patients. Our literature search revealed very few comparable cases to ours, though Athey and colleagues [10] report a case in an older patient treated with splenectomy who died soon after the operation. Therefore, both the incidence and best management of this condition in older people remain unknown.

This case highlights that spontaneous splenic rupture can occur in a previously well, haemodynamically stable patient with no obvious cause and should be suspected in patients with non-specific abdominal pain. In the case of our patient, this was only diagnosed during investigation for deranged liver function tests for which no clear cause was found. There is little evidence in the literature to guide the management of spontaneous splenic rupture in older people.

## Consent

Written informed consent was obtained from the patient's next of kin for publication of this case report and accompanying images. A copy of the written consent is available for review by the Editor-in-Chief of this journal.

## Competing interests

The authors declare that they have no competing interests.

## Authors' contributions

KAM wrote the case report and accompanying discussion. RLS revised the original manuscript and produced the final draft.
